# Weak Broadband Electromagnetic Fields are More Disruptive to Magnetic Compass Orientation in a Night-Migratory Songbird (*Erithacus rubecula*) than Strong Narrow-Band Fields

**DOI:** 10.3389/fnbeh.2016.00055

**Published:** 2016-03-22

**Authors:** Susanne Schwarze, Nils-Lasse Schneider, Thomas Reichl, David Dreyer, Nele Lefeldt, Svenja Engels, Neville Baker, P. J. Hore, Henrik Mouritsen

**Affiliations:** ^1^Institut für Biologie und Umweltwissenschaften, Carl von Ossietzky Universität OldenburgOldenburg, Germany; ^2^Research Centre for Neurosensory Sciences, University of OldenburgOldenburg, Germany; ^3^Department of Chemistry, University of Oxford, Physical and Theoretical Chemistry LaboratoryOxford, UK

**Keywords:** radical pair mechanism, magnetoreception, time-dependent electromagnetic fields, narrow-band electromagnetic field, magnetic compass, bird orientation

## Abstract

Magnetic compass orientation in night-migratory songbirds is embedded in the visual system and seems to be based on a light-dependent radical pair mechanism. Recent findings suggest that both broadband electromagnetic fields ranging from ~2 kHz to ~9 MHz and narrow-band fields at the so-called Larmor frequency for a free electron in the Earth’s magnetic field can disrupt this mechanism. However, due to local magnetic fields generated by nuclear spins, effects specific to the Larmor frequency are difficult to understand considering that the primary sensory molecule should be organic and probably a protein. We therefore constructed a purpose-built laboratory and tested the orientation capabilities of European robins in an electromagnetically silent environment, under the specific influence of four different oscillating narrow-band electromagnetic fields, at the Larmor frequency, double the Larmor frequency, 1.315 MHz or 50 Hz, and in the presence of broadband electromagnetic noise covering the range from ~2 kHz to ~9 MHz. Our results indicated that the magnetic compass orientation of European robins could not be disrupted by any of the relatively strong narrow-band electromagnetic fields employed here, but that the weak broadband field very efficiently disrupted their orientation.

## Introduction

During the last few decades there have been many investigations of how night-migratory birds can use the Earth’s magnetic field for compass orientation and what the underlying sensory processes are (Mouritsen and Hore, 2012; Holland, 2014; Mouritsen, 2015). It is known that migratory birds have an inclination compass, which means that they do not differentiate between north and south but between poleward and equatorward directions (Wiltschko and Wiltschko, 1972, 1995). Thus, they use the angle of the magnetic field lines relative to the Earth’s surface rather than the polarity of the magnetic field. But how do birds sense the direction of the Earth’s field and use it for orientation?

Two main magnetoreception hypotheses are currently discussed. One involves a trigeminal-nerve-related, magnetite (Fe_3_O_4_)-based magnetoreceptor in the upper beak (Kirschvink and Gould, 1981; Winklhofer et al., 2001; Fleissner et al., 2003; Falkenberg et al., 2010). Even though recent studies questioned the existence of such a receptor in the upper beak of pigeons (Mouritsen, 2012; Treiber et al., 2012, 2013), the ophthalmic branch of the trigeminal nerve does seem to be involved in magnetoreception probably related to the birds’ magnetic map sense (Mora et al., 2004; Heyers et al., 2010; Kishkinev et al., 2013; Lefeldt et al., 2014; Kishkinev et al., 2015).

The other hypothesis involves a light-dependent radical-pair-mechanism, working as a chemical sensor for magnetic compass orientation in birds (Schulten et al., 1978; Ritz et al., 2000, 2010; Rodgers and Hore, 2009). It is suggested that a photo-induced electron transfer reaction generates radical pairs which can exist in singlet or triplet electronic spin-states whose coherent interconversion depends on the direction of the Earth’s magnetic field, forming the basis of a magnetic compass (Schulten et al., 1978; Schulten, 1982; Timmel et al., 1998; Ritz et al., 2000; Kavokin, 2009; Rodgers and Hore, 2009; Lau et al., 2010; Solov’yov et al., 2010). Directional sensitivity of a radical pair sensor requires that the electron spin in at least one of the radicals interacts anisotropically (directionally dependent) with magnetic nuclear spins (hyperfine interactions; Ritz et al., 2000; Cintolesi et al., 2003; Efimova and Hore, 2008). The magnetic sensitivity is also influenced by the radical-pair lifetime (Ritz et al., 2000; Efimova and Hore, 2008; Solov’yov et al., 2014); it has been assumed that lifetimes of ~1 μs are realistic for biological systems and that ~1 μs might be close to optimum for sensing the direction of the Earth’s magnetic field (Ritz et al., 2000; Rodgers and Hore, 2009; Gauger et al., 2011). The only class of organic photoreceptors found in vertebrates and known to form long-lived radical pairs upon photo-excitation are cryptochrome proteins (Ritz et al., 2000; Giovani et al., 2003; Liedvogel et al., 2007b; Efimova and Hore, 2008; Solov’yov and Schulten, 2012; Solov’yov et al., 2012). Cryptochromes have been found in the avian retina (Mouritsen et al., 2004; Möller et al., 2004; Liedvogel and Mouritsen, 2010; Nießner et al., 2011; Bolte et al., 2016), and birds and amphibians are known to require light for magnetic compass orientation (Phillips and Borland, 1992; Wiltschko et al., 1993; Engels et al., 2012).

Furthermore, it has been shown that the retinal ganglion cells in the eyes and a forebrain area named Cluster N are highly active when birds use magnetic compass information for orientation behavior (Mouritsen et al., 2004, 2005; Liedvogel et al., 2007a; Zapka et al., 2009). Cluster N is a small part of the visual wulst, which receives its input from the eyes via the thalamofugal visual pathway (Heyers et al., 2007). When Cluster N is inactivated, night-migratory songbirds can no longer use their magnetic compass, whereas their sun and star compasses still function normally (Zapka et al., 2009). Magnetic compass information is therefore processed in Cluster N. Since Cluster N is part of the visual system, this is very strong evidence that the magnetic compass is light-dependent, that the primary sensor must be located in both eyes (Hein et al., 2010, 2011; Engels et al., 2012), and that birds perceive magnetic compass information as a visual impression via the thalamofugal pathway (Ritz et al., 2000; Mouritsen and Hore, 2012; Mouritsen et al., 2016).

In an attempt to test critically whether the magnetic compass of birds is based on a radical pair process, Ritz et al. (2004) investigated the magnetic orientation ability of European robins exposed to time-dependent electromagnetic fields. They found that the birds were disoriented when exposed to either a broadband field (0.1–10 MHz) or a narrow-band electromagnetic field (7 MHz; Ritz et al., 2004). Subsequently (Ritz et al., 2009), it was reported that European robins, when tested in Frankfurt (Germany), were particularly sensitively disoriented by a narrow-band electromagnetic field oscillating at the “Larmor frequency”. For organic radicals, the Larmor frequency [= 1.315 MHz in Frankfurt (Ritz et al., 2009) and 1.363 MHz in Oldenburg, where the experiments reported below were performed] is the energy of the interaction of an electron with the local geomagnetic field divided by Planck’s constant, *h*. To explain why a magnetic field at the Larmor frequency should have a much more pronounced effect on the birds than one of the same intensity at half or double that frequency, it was suggested that one of the two radicals in the magnetically sensitive radical pair had to be devoid of hyperfine interactions (Ritz et al., 2009). In practice this means a radical in which there are no hydrogen or nitrogen atoms in the vicinity of the molecular orbital that contains the unpaired electron. If there are no hyperfine interactions in a radical then the only magnetic field experienced by its electron would be the geomagnetic field, resulting in a unique energy-level-splitting equal to *h* times the Larmor frequency. However, if the electron also experiences magnetic fields from surrounding nuclei, there will be many energy-level-splittings corresponding to a variety of frequencies. In the latter case one would not expect a particularly sensitive response to a Larmor frequency magnetic field. The difficulty with this interpretation of the Frankfurt data is that almost every biologically plausible organic radical has several hydrogens and/or nitrogens. The only radical without hyperfine interactions that has been discussed in this context is superoxide (${\text{O}}_{2}^{•-}$; Maeda et al., 2008; Ritz et al., 2009; Solov’yov and Schulten, 2009; Müller and Ahmad, 2011). Although attractive in some respects, other properties of ${\text{O}}_{2}^{•-}$ make it almost certainly unsuitable as a participant in a radical pair magnetoreceptor (Hogben et al., 2009). The observation of an apparent resonance at the Larmor frequency (Ritz et al., 2009) therefore remains puzzling. Consequently, we decided that independent replication of Ritz et al. (2009) was essential to guide future directions of research into radical-pair-based magnetoreception in birds.

One of the difficulties in investigating the influence of time-dependent magnetic fields is that most experiments have been performed in big cities and/or in the neighborhood of universities where irregular and uncontrollable anthropogenic time-dependent electromagnetic fields are omnipresent (Engels et al., 2014). Consequently, to perform proper experiments with birds exposed to time-dependent magnetic fields, it is absolutely essential to design and construct a testing laboratory in which the external time-dependent fields are effectively and reliably excluded, while the static geomagnetic field is left undisturbed. Otherwise, we cannot be sure that the birds were only exposed to the intended narrow-band electromagnetic fields and/or weak broadband fields.

Therefore, to do this study properly, we constructed a unique laboratory, which allows for unprecedented control of static and time-dependent fields. In this laboratory, the static geomagnetic field was completely natural, homogeneous and undisturbed while time-dependent magnetic fields in the frequency range from about 10 kHz to 1 GHz were attenuated by a factor of at least 10^5^. The aim of the present study is to use this newly constructed electromagnetically silent environment to test whether the magnetic compass orientation behavior of night-migratory European robins is really affected by strong narrow-band electromagnetic fields and/or weak broadband fields.

## Materials and Methods

### Newly Constructed Wooden Laboratory

**Figure 1:**
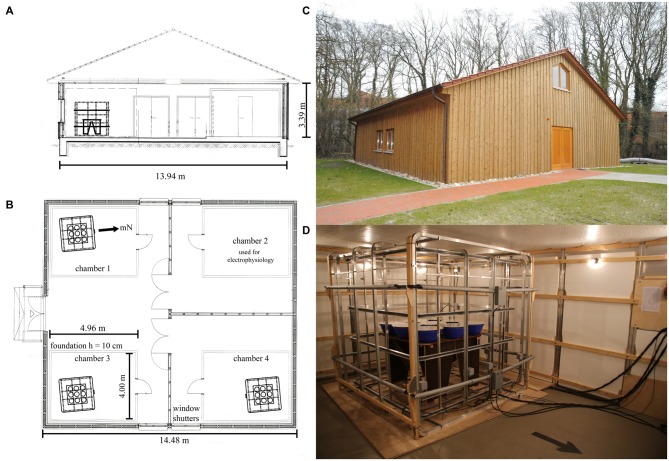
**Ground plan of the new wooden laboratory.** The materials used to build the laboratory were strictly non-magnetic. Four experimental chambers, which very efficiently screen time-dependent electromagnetic fields, are placed within the building. One double-wrapped, three-dimensional Merritt four-coil system with average coil diameters of ~2 m (Zapka et al., 2009; Hein et al., 2010; Engels et al., 2014) and a pair of 2.2 m *z*-axis RF-coils in Helmholtz-configuration was placed within three of these four chambers. **(A)** Cross-section. **(B)** Floor plan. **(C)** Photo of the new wooden laboratory from the outside. **(D)** Photo of one of the coil setups placed inside each of the three chambers. The photo also shows the nine Emlen funnels in their holders, which were placed on top of the wooden table in the center of the coil system.

We constructed a purpose-built laboratory on the University of Oldenburg campus (Figure 1). The 188 m^2^ wooden house (outside measurements 14.48 m × 13.94 m × 3.39 m) is built completely free from ferromagnetic materials. It consists of three rooms and rests on five separate foundations, one for each of the aluminum-screened chambers (see below) and one for the access areas and the walls. This construction ensures that vibrations from people walking within the building are not transmitted to the experimental chambers. The foundations are made of concrete with non-metallic reinforcement. The walls, attic floor, and roof are all constructed of wood and all connections, nails etc. are made from non-magnetic stainless steel, copper, and aluminum. Each of the three rooms contains one or two aluminum-based, electromagnetically screened chambers (S101, ETS Lindgren, Germany) measuring 4.96 m × 4.00 m × 2.48 m (Figure 1). Each chamber is placed on an extremely level (to within ±5 mm in 10 m and ±1 mm in 1 m) surface with its own separate foundation. This is essential to ensure that the panels from which the chamber is built remain in perfect contact with each other and do not become leaky over time. Within the whole laboratory containing four chambers, the coil systems and thus the orientation funnels were placed as far as possible from each other, consistent with the experimenters being able to walk around each of the coil systems in order to place birds into the funnels from all sides (see Figure 1). The distance between the coil systems was maximized so that the static fields generated by any given coil system within one chamber would interfere as little as possible with the geomagnetic field and/or the static fields generated by the coils within the other chambers (the static fields generated by a coil system attenuate with the third power of the distance from the coil system).

**Figure 2:**
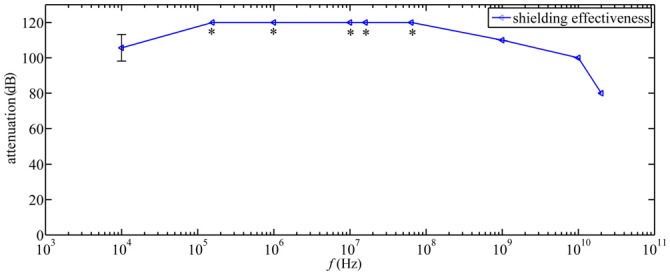
**Shielding effectiveness measurements in experimental chambers at different frequencies in accordance to the IEEE-299 standard (IEEE, 1997).** *indicates that the shielding effectiveness was higher than the largest measurable value of 120 dB.

The chambers are constructed as Faraday cages so that static magnetic fields, such as the geomagnetic field, penetrate the aluminum screens unaltered, whereas time-dependent electromagnetic fields are strongly attenuated. The screening efficiency of the chambers was 10^5^ at 10 kHz and >10^6^ at frequencies above 150 kHz (Figure 2). The electric and magnetic screening efficiency of the chambers was measured immediately after being constructed and multiple times over the following years (for details of the used equipment, see below under “Time-Dependent Magnetic Fields” Section). The four chambers are grounded via individual grounding rods all connected to one single ground-electrode loop integrated into the concrete base of the laboratory. Furthermore, after spring season 2013, all the electronic equipment associated with each chamber and involved in generating its time-dependent electromagnetic fields was grounded to an independent 8 m deep grounding rod (i.e., four grounding rods in total, one for each chamber). Inside the shielded chambers, the four walls are reinforced with wooden slats to which are fixed plates of styro-foam, which are used to thermally insulate the chambers and to prevent any potential low frequency vibrations of the walls. Air from outside the building is blown into the chambers through a honeycomb ventilation module, so that the odors and temperatures inside the chambers are similar to those outside.

All items of electric equipment used for the experiments (signal generators, power supplies, etc.) were placed outside the chambers and were, as mentioned above, electrically isolated from the equipment of the other chambers through the separate grounding rods. All electrical communications into the chambers are either passed through special filters, via a forwarding panel bearing BNC and N1 connectors, or are converted to optical signals and sent through light guides penetrating the honeycomb ventilation module before being converted back into electrical signals inside the chambers. In this way, no electromagnetic interference from outside can enter the chambers.

### Test Animals

A total number of 91 (spring 2012 = 32; spring 2013 = 21; autumn 2013 = 17; spring 2014 = 21) European robins (*Erithacus rubecula*) were tested in this study. All birds were caught and tested on the campus of the University of Oldenburg, Germany. The birds were housed indoors in individual cages in a windowless room with a light regime matching the local photoperiod. All procedures were performed in accordance with local and national guidelines for the use of animals in research and were approved by the Animal Care and Use Committees of the Niedersächsisches Landesamt für Verbraucherschutz und Lebensmittelsicherheit (LAVES), Oldenburg, Germany (protocol log numbers: 33.12-42502-4-07/1422 and 33.12-42502-04-13/1065) and the USAMRMC Animal Care and Use Review Office (ACURO).

### Static Magnetic Fields

Experimental magnetic fields were produced by double-wrapped, three-dimensional Merritt four-coil systems (Kirschvink, 1992) with average coil diameters of ~2 m (Zapka et al., 2009; Hein et al., 2010; Engels et al., 2014). Each of the three axes was driven by a separate constant current power supply (BOP 50-2M or BOP 50-4M, Kepco Inc., Flushing, NY, USA). All experiments were performed using nine Emlen Funnels (see below) placed within the central space of the coils in which the heterogeneity of the static magnetic field was less than 1% (the setup is illustrated in Figure 20.2a in Mouritsen, 2013). Before the start of each experiment, the magnetic field was measured in the center and at the edges of the experimental space within the coils, where nine orientation cages (modified Emlen funnels; Emlen and Emlen, 1966) were placed for the experiments. Birds were tested in two different magnetic field conditions: in a field virtually identical to the natural geomagnetic field (NMF) in Oldenburg (magnetic field strength (flux density) = 48,600 nT ± 240 nT [standard deviation, SD]; inclination = 67.3° ± 0.4° [SD]; horizontal direction 360° ± 0.1° [SD]), and in a magnetic field with magnetic North (mN) turned 120° counter-clockwise (changed magnetic field, CMF: magnetic field strength (flux density) = 48,600 nT ± 250 nT [SD]; inclination = 67.4° ± 0.3° [SD]; horizontal direction = −120° ± 2° [SD]). For the NMF condition, the same current that was needed to create the CMF condition was sent through the two subsets of coil windings but in opposite (antiparallel) directions, so that the effective changes in the NMF strength (flux density) were less than 10 nT. This means that, in the NMF condition, the real Earth’s magnetic field to which the laboratory is permeable was the only magnetic field available to the birds and measurable for us.

### Time-Dependent Magnetic Fields

**Figure 3:**
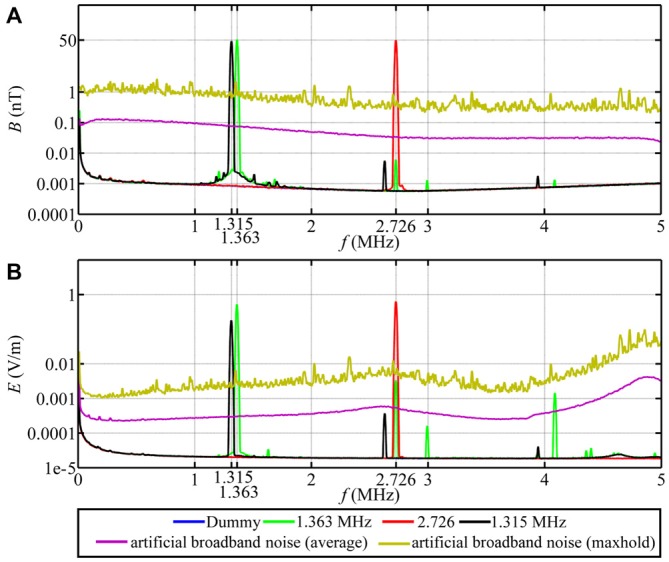
**Magnetic and electric field measurements of test conditions in spring 2012, autumn 2013 and spring 2014.** Traces in **(A,B)** show, respectively, the magnetic (*B*) and electric (*E*) components of the narrow-band electromagnetic fields and the artificial broadband noise as a function of frequency (*f*). The narrow-band magnetic fields had a peak intensity of 48 nT. The intensity of the broadband noise is shown twice: the purple line is the average (root mean square) intensity and the yellow line is the 40 min max-hold intensity and is directly comparable to the spectra shown in Engels et al. (2014).

**Figure 4:**
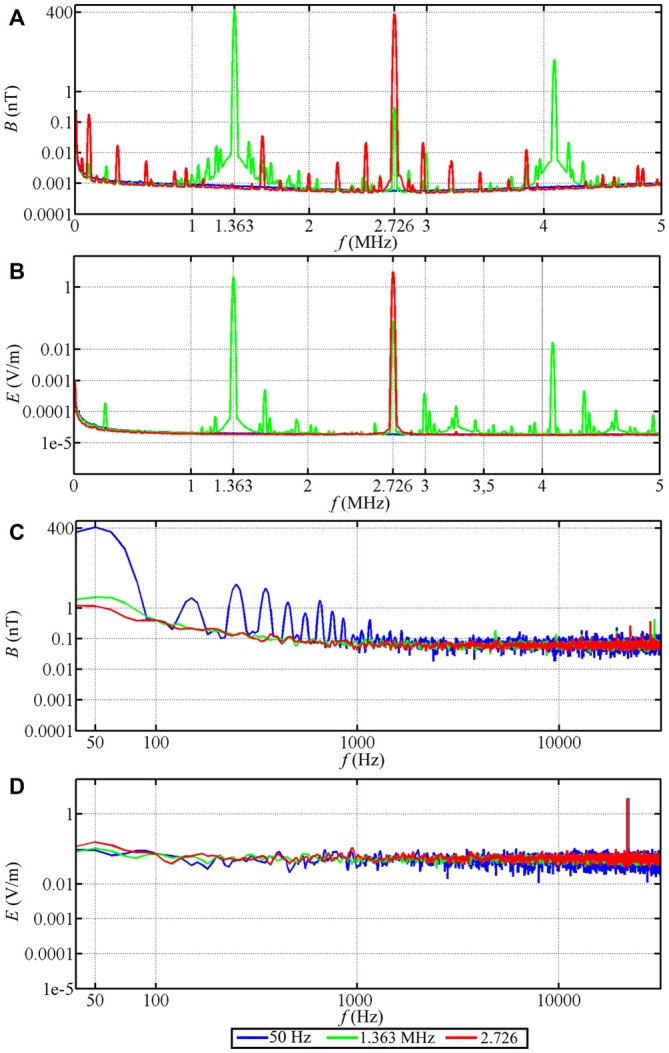
**Magnetic and electric field measurements of test conditions in spring 2013.** Traces in **(A–D)** show, respectively, the magnetic (*B*) and electric (*E*) component of the narrow-band electromagnetic fields in the range from 10 kHz to 5 MHz **(A,B)** and in the range from 40 Hz to 32 kHz **(C,D)**. The peak intensity in each condition was ~400 nT.

For the time-dependent magnetic field exposure experiments, the desired center frequency or frequency range was set for each experimental chamber. The intensity of the narrow-band electromagnetic fields was fine-tuned using a spectrum analyzer (Aaronia AG, SPECTRAN NF-5030, Germany). This was done every day before the experiments were started. Additional measurements of the magnetic and electric components of the time-dependent magnetic fields were performed using a signal analyzer (Rohde and Schwarz, FSV 3 Signal and Spectrum Analyzer, 10 Hz–3.6 GHz, Germany). The magnetic components were measured with a calibrated passive loop antenna (ETS Lindgren, Model 6511, 20 Hz–5 MHz, Germany) in the range between 10 kHz and 10 MHz. The electric components were measured with a calibrated active bi-conical antenna (Schwarzbeck Mess-Electronik, EFS 9218, 9 kHz–300 MHz, Germany). Two types of measurements were made: the signal analyzer was set to “average” with a resolution bandwidth of 10 kHz to make the measurements comparable with Ritz et al. (2009) or to “max-hold” with a resolution bandwidth of 10 kHz to allow the broadband noise employed in this study to be compared with the measurements of Engels et al. (2014). Therefore, strictly speaking the correct magnetic field unit is not nT as indicated on the Figures 3, 4, but nT/√10kHz. For the low-frequency range below 32 kHz, the magnetic components were measured with a calibrated 100 cm^2^ magnetic field probe (EFA-300 system, Narda Safety Test Solutions, Germany) and the electric components were measured with a calibrated electric field unit (EFA-300 system, Narda Safety Test Solutions, Germany). The antennas were connected to an EFA-300 hand-held signal analyzer which was also set to “average” or “max-hold” (Engels et al., 2014). The traces in Figures 3, 4 are based on 10,000 equally spaced measurement points between 10 kHz and 10 MHz. By “narrow-band” we mean that a monochromatic (e.g., 1.363 MHz) voltage applied to the coils generates a field (e.g., ~48 nT) with a bandwidth of ~0.30 MHz around the center frequency together with weak side-bands in the range 1.213 MHz to 1.511 MHz and weak components at the harmonic frequencies, see Figure 3A, green trace. For further information on the time-dependent magnetic fields and how we calculated the summed magnetic field intensity in the range up to 10 MHz see Table 1 and Engels et al. (2014). The setting, control and adjustment of the specific time-dependent fields were done by investigators who neither handled the birds nor evaluated the orientation data. The researchers who performed the experiments had no way of knowing what magnetic conditions the birds were exposed to in any given test. All experiments and evaluations were thus performed double blind in order that the experimenters could not be subconsciously influenced by any expectation of the outcomes.

**Table 1:** **Measured time-dependent magnetic field intensities**.

Migratory	Frequency	Peak intensity/nT	Summed magnetic intensity^2^/nT (10 kHz–10 MHz)	Magnitude ofseason experimental effect^1^
Spring 2012	1.363 MHz	~48	80	+
	2.726 MHz	~48	75	+
	Dummy	~48	1	−
Spring 2013	1.363 MHz	~400	799	−
	2.726 MHz	~400	598	−
	50 Hz	~400	1000	−
Autumn 2013	1.315 MHz	~48	71	−
	Dummy	~48	1	−
	Broadband noise	varies with frequency, see Figure 3	35	+++
			5 mT^3^	
Spring 2014	1.315 MHz	~48	80	−
	Dummy	~48	1	−

*^1^−, no effect; +, possibly a small effect; +++, strong effect. ^2^The average total magnetic field intensity (more precisely the magnetic flux density, *B*) in the frequency range between 10 kHz and 10 MHz was calculated using the following equation:*
$$B(\Delta f)=\frac{1}{N}\frac{\Delta f}{\Delta f_{0}}\sum_{i}B_{i}(f_{i},\Delta f_{0})$$
*B(Δf) denotes the total magnetic flux density in the bandwidth of interest, Δf = 10 MHz–10 kHz = 9990 kHz, and B_i_(f_i_, Δf_0_) are the magnetic flux densities at the *N* different frequency values f_i_ (every 1 kHz between 10 kHz and 10 MHz, i.e., *N* = 9990) for a resolution bandwidth Δf_0_ which equals 10 kHz here. Expressed in words, B(Δf) = (the sum of the magnetic field intensity values/# of values) × (frequency range size/resolution bandwidth), in our case: (the sum of the magnetic field intensity values/9990) × (9990 kHz/10 kHz) for the total frequency range from 10 kHz to 10,000 kHz. The “average” values allow direct comparison with the narrow-band fields used in Ritz et al. (2009). ^3^Accumulated maximum field strength for the whole range from 10 kHz to 10 MHz, measured over a period of 40 min with the detector set to “max-hold”. In other words, the maximum possible intensity that would occur if all the strongest disturbances at all frequencies, measured over 40 min period, would occur at one point in time. It must be stressed that the total magnetic field strength indicated when calculated by adding up max-hold values strongly almost certainly overestimates the highest total magnetic field strength that will have occurred at any specific point in time. The “max-hold” value allows for comparison with the study of Engels et al. (2014)*.

We used a pair of copper Helmholtz coils (outside dimension 2.14 m × 2.14 m), mounted horizontally around the Merritt coil system, to expose the birds to time-dependent magnetic fields with one of four narrow-band electromagnetic fields or to broadband noise covering the frequency range from ~2 kHz to ~9 MHz. With this arrangement, the oscillating fields were vertical, i.e., at an angle of 23° relative to the static field (67° inclination). In spring 2012, the narrow-band electromagnetic fields were produced by a signal generator (RIGOL^®^ DG 1022, Germany) and an amplifier (TOMCO, CW RF Amplifier System 50 W, UK), and passed through a forwarding panel (see above) to a pair of tuning boxes linked to the copper Helmholtz coils. Each time-dependent magnetic field had its individual set of tuning boxes. One set of additional boxes operated as a dummy condition, where no current was sent through the copper Helmholtz coils and thus no time-dependent fields were created around the Emlen funnels. The dummy tuning boxes forwarded the time-dependent fields to so-called dummy loads. These dummy loads were fixed under the tuning boxes and converted the signal into thermal energy, which was released into the environment (the temperature change of the dummy loads was less than 4°C). The tuning boxes were fitted with blinded codes, so that only the persons who adjusted the time-dependent fields knew to which field the birds were exposed. The persons who performed the experiments and evaluated the data did not know what the symbols meant. The symbols were only unblinded after all data had been collected and analyzed.

After the migratory season in the autumn of 2012, we slightly improved the arrangement of the equipment. The signal generator, amplifier and a blinding box were all still placed outside the experimental chamber but were now placed inside a wooden box lined with aluminum plates to further reduce any interference caused by the equipment itself. The blinding box was inserted after the signal generator and forwarded the generated signal either to the dummy loads (dummy condition), to the amplifier and then to the tuning boxes and copper Helmholtz coils (in case of the 1.363 MHz, 2.726 MHz and 1.315 MHz conditions), or directly through the forwarding panel to the tuning boxes and copper Helmholtz coils (50 Hz and broadband noise conditions).

The orientation of the birds was tested under each of the following five exposure conditions: 1.363 MHz, 2.726 MHz, 50 Hz, and 1.315 MHz narrow-band electromagnetic fields, and broadband noise. In addition, there were various dummy conditions in which no time-dependent fields were present within the experimental area (see Table 1 for intensities). The choice of frequencies was determined by the local geomagnetic fields: 1.363 MHz and 2.726 MHz are the Larmor frequency and twice the Larmor frequency in Oldenburg and 1.315 MHz is the Larmor frequency in Frankfurt (Ritz et al., 2009). The 50 Hz frequency was chosen as an exposure condition because it should effectively be a static field to a radical-pair with a microsecond lifetime, and because it matches the prevailing frequency in electric circuits in Germany. The ~48 nT peak intensity corresponds to one used by Ritz et al., 2009 and ~400 nT was chosen as the maximal intensity that our equipment could reliably produce.

### Behavioral Experiments

Each autumn and spring, we started our experiments 1 to 2 weeks before the regular migratory period for European robins to accustom the birds to the handling procedure and the set-up. During this phase, the birds’ orientation tended to be more random or inactive (see below). As a sign that the migratory season had started, the birds’ orientation improved and was aligned in the appropriate migratory direction. This was taken as a sign that the birds were in migratory mood. The birds were then pre-tested in the NMF and CMF conditions to ensure that they used the Earth’s magnetic field to orient in their appropriate migratory direction. In spring the predicted migratory direction is north-east (~350°−80° NMF; ~230°−320° CMF) and in autumn south-west (~180°−270° NMF; ~60°−150° CMF). These pre-tests were performed in our older wooden huts, which were shielded from external time-dependent electromagnetic fields by approximately two orders of magnitude (Engels et al., 2012, 2014).

All active and properly oriented birds participated in the following critical tests in the new laboratory, where they were exposed to either a NMF or a CMF static field as well as specific time-dependent electromagnetic fields.

All behavioral experiments were conducted in the following way. One hour before sunset and the start of the tests, the birds were placed outdoors in wooden transport boxes to enable them to see twilight cues and parts of the evening sky to give them the possibility to calibrate their magnetic compass (Cochran et al., 2004; Muheim et al., 2006a,b, 2009). The same procedure was followed independent of the weather. Immediately thereafter (at sunset), the birds were placed in the modified aluminum Emlen funnels (35 cm diameter, 15 cm high, walls 45° inclined; Emlen and Emlen, 1966). During testing, the experimental chamber was illuminated with dim light (2.5 ± 0.25 mW m^−2^) produced by incandescent bulbs (spectrum in Zapka et al., 2009).

Nine birds per night were tested simultaneously in each hut or chamber. Two rounds of tests took place each night, the second starting approximately 1.5 h (±10 min) after the first. During the second round, each bird was tested in a different chamber or at a different funnel position but under the same static magnetic field condition (NMF or CMF) and in the same time-dependent electromagnetic field condition (dummy, 50 Hz, 1.315 MHz, 1.363 MHz, 2.726 MHz or broadband). This procedure prevented the birds from remembering or transferring any possible non-magnetic cues from one test to the next. Furthermore, no bird was subjected to the same test condition for more than two successive nights.

The Emlen funnels were lined with scratch-sensitive paper (Mouritsen et al., 2009) on which the birds left scratches as they moved within the funnels. From these scratches, the mean orientation of the birds could be determined. The overlap point of the paper was oriented towards north, east, south or west with the direction varied randomly from chamber to chamber and night to night. After testing, the papers were evaluated relative to the overlap point. Hence, the evaluators did not know to which of the four cardinal directions the overlap point corresponded, and they were also blind to the experimental conditions. In this way, we could exclude that the results were subjectively biased. The direction of the overlap point was only revealed after the scratches had been evaluated. At the start of each experiment, the time-dependent electromagnetic field was only turned on after the experimenter had left the chamber, a procedure required by safety regulations. In spring 2013 (13–30 March), a third round of tests was added because, judged by the scratches on the papers, the birds were as active in the second test round as in the first (which was usually not the case).

### Orientation Data Analysis

Two researchers independently visually estimated each bird’s mean direction from the distribution of the scratches (Mouritsen, 1998). The evaluation of the papers was blinded, i.e., the evaluators did not know the magnetic field condition experienced by the birds, the direction of the overlap point of the paper (see above), or the time-dependent electromagnetic field condition. If the two independently determined mean directions differed more than 30°, or if at least one observer considered the distribution of the scratches as random, a third independent evaluator was asked to determine the mean direction. If this mean direction was similar to one of the first two, and if all three observers could agree with this mean direction, the mean of the two similar directions was taken as the mean orientation direction of the bird. If the three evaluators could not agree on one mean direction, the bird’s heading was regarded as random and the paper was excluded from the analyses (6% out of a total of 4098 individual tests). When the birds were placed in the Emlen funnels and taken out again shortly thereafter, they could leave up to 80 scratches while they tried to escape in random directions. Based on this observation, and in contrast to the threshold of 30 scratches used in the past (e.g. Zapka et al., 2009; Hein et al., 2010), we excluded all papers with fewer than 100 scratches (24% of all papers; Engels et al., 2012, 2014). Additionally, all papers with a bimodal direction were excluded (2% of all papers). To calculate the average mean heading of an individual bird in a given experimental condition, the mean direction of all its oriented tests was calculated by addition of unit vectors in each of the mean directions of the individual tests. The group mean vectors were calculated by identical vector addition of these individual mean directions followed by division by the number of birds tested in the given condition. The significance of the group mean vector was tested using the Rayleigh-test (Batschelet, 1981). Differences in group mean orientations between birds tested in different magnetic field conditions were tested by the Mardia-Watson-Wheeler-test (MWW; Batschelet, 1981).

## Results

**Figure 5:**
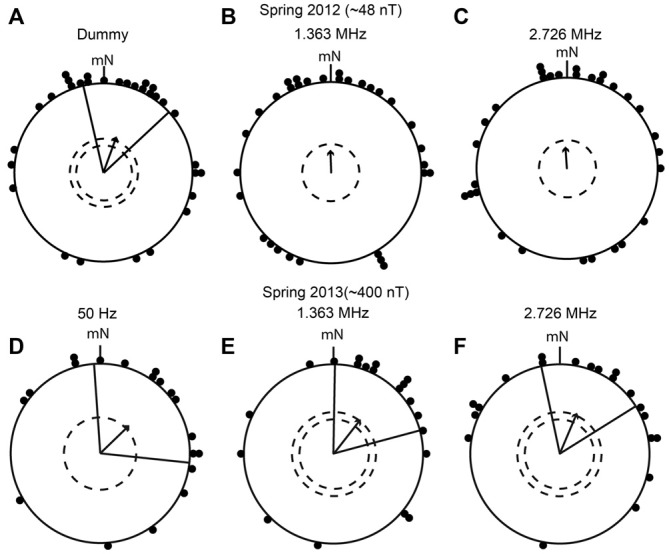
**European robins can orient in their appropriate migratory directions when they are exposed to narrow-band time-dependent magnetic fields. (A–F):** The results of orientation cage experiments on European robins. **(A)** Dummy condition in which the birds were not exposed to time-dependent magnetic fields (spring 2012, mean direction 17° ± 30° (95% confidence interval), *r* = 0.43, *p* < 0.01, *N* = 31). **(B)** Birds exposed to 1.363 MHz with a peak intensity of 48 nT (spring 2012, mean direction 358°, *r* = 0.25, *p* = 0.138, *N* = 31). **(C)** Birds exposed to 2.726 MHz with a peak intensity of 48 nT (spring 2012, mean direction 356°, *r* = 0.24, *p* = 0.179, *N* = 30). **(D)** Birds exposed to 50 Hz with a peak intensity of ~400 nT (spring 2013, 46° ± 50°, *r* = 0.45, *p* < 0.05, *N* = 19). **(E)** Birds exposed to 1.363 MHz with a peak intensity of ~400 nT (spring 2013, 38° ± 37°, *r* = 0.48, *p* < 0.01, *N* = 20). **(F)** Birds exposed to 2.726 MHz with a peak intensity of ~400 nT (spring 2013, 23° ± 35°, *r* = 0.49, *p* < 0.01, *N* = 20). Each dot represents the mean heading of an individual bird, the arrows indicate the group mean vector, the radial lines flanking the group mean vector indicate the 95% confidence interval for the group mean direction and the dashed circles represent the significance levels (*p* < 0.05 and *p* < 0.01) according to the Rayleigh test. mN, magnetic North.

In four migratory seasons since spring 2012, the birds were tested in time-dependent electromagnetic fields at five narrow-band frequencies ranging from 50 Hz to 2.726 MHz with intensities of either ~48 nT or ~400 nT, and in one broadband field covering the range from ~2 kHz to ~9 MHz. In the first season (spring 2012), the birds were exposed to narrow-band electromagnetic fields with frequencies of 1.363 MHz, 2.726 MHz at an intensity of ~48 nT, and to a 1.363 MHz dummy condition. They were only tested in the NMF. The birds were significantly oriented in their appropriate migratory direction in the dummy condition (mean direction 17° ± 30° [95% confidence intervals], *r* (length of the mean group vector) = 0.43, *p* < 0.01, *N* = 31; Figure 5A), and the same birds showed a non-significant, but clear, tendency to orient in the correct migratory direction when exposed to a narrow-band electromagnetic field at 1.363 MHz (mean direction 358°, *r* = 0.25, *p* = 0.138, *N* = 31; Figure 5B), and at 2.726 MHz (mean direction 356°, *r* = 0.24, *p* = 0.179, *N* = 30, Figure 5C). The Mardia-Watson-Wheeler test showed no significant difference between the three conditions (MWW: *W* = 1.807, *p* = 0.771). This suggests that either the birds were influenced to some extent by the narrow-band fields at both the Larmor frequency and at double the Larmor frequency but fundamentally were able to orient, or that their orientation was disrupted and the orientation towards north was a coincidence.

To clarify this, in spring 2013, we exposed the birds to the same frequencies as in spring 2012 (1.363 MHz and 2.726 MHz), but at an intensity of ~400 nT (instead of ~48 nT), and replaced the dummy condition with an exposure to a 50 Hz field with an intensity of ~400 nT. Our expectation was that the birds would not be affected by a 50 Hz field which changes so slowly that it would be experienced by a radical-pair-based magnetoreceptor as a tiny additional static magnetic field (Timmel et al., 1998; Rodgers and Hore, 2009; Engels et al., 2014; Maeda et al., 2015). The birds showed significant orientation in the appropriate migratory direction relative to mN in all three conditions (50 Hz: 46° ± 50°, *r* = 0.45, *p* < 0.05, *N* = 19; 1.363 MHz: 38° ± 37°, *r* = 0.48, *p* < 0.01, *N* = 20; 2.726 MHz: 23° ± 35°, *r* = 0.49, *p* < 0.01, *N* = 20; Figures 5D–F). None of the three narrow band electromagnetic fields seemed to significantly affect the birds’ magnetic compass orientation capabilities, despite the much higher intensity (~400 nT). Furthermore, there were no significant differences in the directional distributions between the three conditions (MWW: *W* = 3.934, *p* = 0.415). Considering these results, the birds tested in spring 2012 under the same fields, but with ~48 nT intensity, would probably have been found to be significantly oriented towards the north (like the controls) if we had had the opportunity to do more tests.

**Figure 6:**
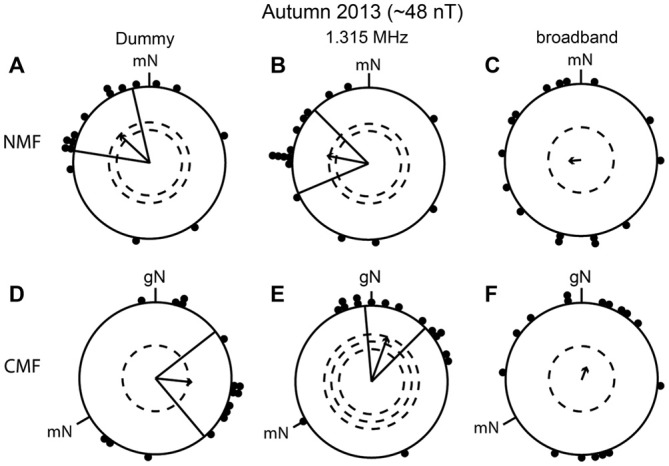
**Broadband electromagnetic noise disrupts the birds’ magnetic orientation capabilities whereas a narrow-band field at 1.315 MHz does not.** The birds were able to orient in the dummy condition in the natural geomagnetic field (NMF), **(A)**, mean direction 313° ± 34°, *r* = 0.54, *p* < 0.01, *N* = 16 and the changed magnetic field (CMF), **(D)**, mean direction 96° ± 44°, *r* = 0.49, *p* < 0.05, *N* = 16 and when exposed to 1.315 MHz (NMF, **(B)**, mean direction 281° ± 34°, *r* = 0.56, *p* < 0.01, *N* = 16; CMF, **(E)**, 1.315 MHz: mean direction 20° ± 25°, *r* = 0.62, *p* < 0.001, *N* = 16). In contrast, the same birds were disoriented when exposed to artificial broadband noise (~2 kHz to ~9 MHz), NMF: **(C)**, mean direction 265°, *r* = 0.17, *p* = 0.603, *N* = 16; CMF: **(F)**, 21°, *r* = 0.19, *p* = 0.557, *N* = 16. All experiments were done in autumn 2013. For a description of the circular diagrams, see the legend to Figure 5. gN, geographic North.

In the light of the results in spring 2012 and 2013, we decided in autumn 2013 to test whether narrow-band electromagnetic fields at 1.315 MHz and ~48 nT, which were reported to disrupt the compass orientation of European robins in Frankfurt (Thalau et al., 2005; Ritz et al., 2009; Wiltschko et al., 2014), would lead to disorientation of our European robins in Oldenburg. In autumn 2013 we also tested the same birds in a broadband noise-modulated electromagnetic field (~2 kHz to ~9 MHz) and in a 1.315 MHz dummy condition (see above). The artificial broadband noise was similar to that used by Engels et al. (2014). The birds were significantly oriented both in the dummy condition and when exposed to the 1.315 MHz narrow-band electromagnetic field in the NMF condition (dummy: mean direction 313° ± 34°, *r* = 0.54, *p* < 0.01, *N* = 16; 1.315 MHz: mean direction 281° ± 34°, *r* = 0.56, *p* < 0.01, *N* = 16; Figures 6A,B) and also in the CMF condition (dummy: mean direction 96° ± 44°, *r* = 0.49, *p* < 0.05, *N* = 16; 1.315 MHz: mean direction 20° ± 25°, *r* = 0.62, *p* < 0.001, *N* = 16; Figures 6D,E). In contrast, the same birds were disoriented when they were exposed to the ~2 kHz to ~9 MHz broadband field (NMF: mean direction 265°, *r* = 0.17, *p* = 0.63, *N* = 16; Figure 6C; CMF: mean direction 21°, *r* = 0.19, *p* = 0.57, *N* = 16, Figure 6F). The mean headings of the birds in the dummy and 1.315 MHz NMF conditions were not in the expected southwesterly direction (Figures 6A,B). Furthermore, although the mean orientations of the birds tested in the NMF condition in the dummy and 1.315 MHz conditions differed significantly from those in the CMF condition (95% confidence intervals did not overlap; MWW: dummy condition: *W* = 13,874, *p* < 0.001; 1.315 MHz: *W* = 15, 693, *p* < 0.001), the directional shift of the birds’ orientation when mN was rotated counterclockwise by −120° in the CMF condition was significantly different from the expected value of −120° (compare mean direction in Figures 6A,D: a −217° shift; and Figures 6B,E: a −261° shift).

**Figure 7:**
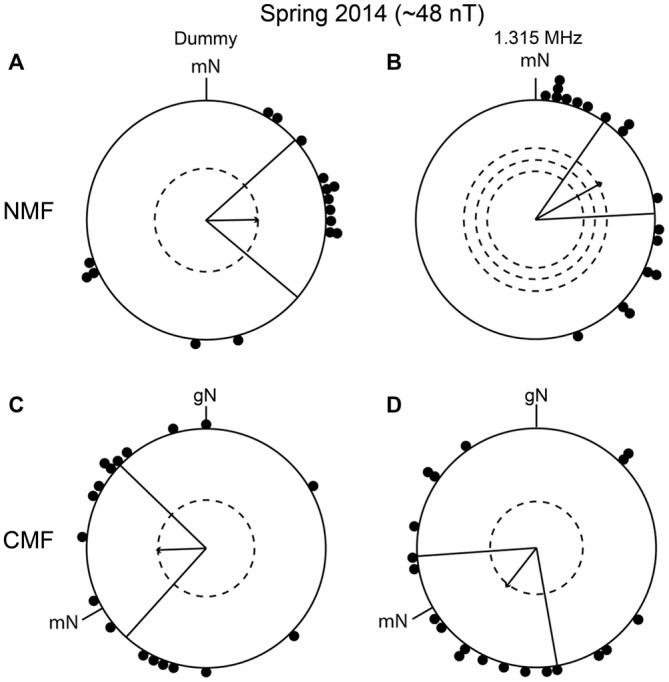
**Replication of the experiments from autumn 2013. (A,C)** The birds’ orientation under the dummy condition in spring 2014. **(B,D)** The orientation of the birds during exposure to 1.315 MHz in spring 2014. The data in **(A,B)** were collected in an unchanged magnetic field (NMF, dummy: mean direction 89° ± 41°, *r* = 0.44, *p* < 0.05, *N* = 16; 1.315 MHz: mean direction 61° ± 26°, *r* = 0.64, *p* < 0.001, *N* = 18). The data in **(C,D)** were collected in a magnetic field turned 120° counterclockwise (CMF, dummy: mean direction 268° ± 46°, *r* = 0.42, *p* < 0.05, *N* = 18; 1.315 MHz: mean direction 218° ± 48°, *r* = 0.42, *p* < 0.05, *N* = 20). For a description of the circular diagrams, see the legend to Figure 5.

To investigate whether the apparently wrongly directed orientation of the birds in autumn 2013 was reproducible, we decided to re-test birds in the same conditions. In spring 2014, the birds were significantly oriented in their appropriate migratory direction in the NMF condition, in the dummy condition and when exposed to a 1.315 MHz, ~48 nT field (dummy: mean direction 89° ± 41°, *r* = 0.44, *p* < 0.05, *N* = 16; 1.315 MHz: mean direction 61° ± 26°, *r* = 0.64, *p* < 0.001, *N* = 18, Figures 7A,B) as well as under the CMF condition (dummy: mean direction 268° ± 46°, *r* = 0.42, *p* < 0.05, *N* = 18; 1.315 MHz: mean direction 218° ± 48°, *r* = 0.42, *p* < 0.05, *N* = 20; Figures 7C,D). The mean migratory direction of the birds differed significantly between NMF and CMF conditions (MWW: dummy: *W* = 11,019, *p* = 0.004; 1.315 MHz: *W* = 18,267, *p* < 0.001) but again did not fit well with the expected −120° shift (compare mean direction in Figures 7A,C: dummy: a −181° shift; and Figures 7B,D: 1.315 MHz: a −203° shift). Currently, we do not understand what these unexpected shifts mean. It is, however, very unlikely that the significant orientation of the birds in the 1.315 MHz fields was caused by an artifact or that they represent “fixed direction responses”, as for example reported for different light intensities (Wiltschko et al., 2000, 2007, 2010), since the birds were significantly oriented towards different directions in spring and autumn and in different directions in the NMF and CMF conditions within any given season.

## Discussion

Our findings indicate that strong, narrow-band electromagnetic fields did not disrupt the magnetic compass of migratory birds. There might have been some disturbance to the operation of the birds’ magnetic compass, since it seems that we would have needed to perform more tests with the birds in order to reach the 5% significance level when they were exposed to the narrow-band fields compared to the dummy conditions in spring 2012. In spring 2012 we performed exactly the same number of tests on all the tested individuals in each of the three applied conditions. Therefore the orientation of the birds (*r*-values and *p*-values) was directly comparable between groups: while the northerly orientation of the birds in the dummy condition was statistically significant (*p* < 0.01, *r* = 0.43), the 1.363 MHz and 2.726 MHz groups only showed strong tendencies towards the north (*p* = 0.138, *r* = 0.25 and *p* = 0.179, *r* = 0.24, respectively). We interpret the spring 2012 results as follows: the birds were fundamentally able to orient towards the north, but were somewhat disturbed by the radio-frequency fields so that the directional scatter increased. Furthermore, the birds shifted their orientation when mN was rotated counterclockwise by −120°, but not always by the expected −120° angle. All of this could indicate that the birds were somewhat affected by the narrow-band electromagnetic fields but that their magnetic compass was not completely impaired. In contrast, the birds’ orientation was completely disrupted by the ~2 kHz to ~9 MHz broadband field, in which the birds showed no tendency to orient towards the appropriate direction in any of the static fields and all the *r*-values were very low.

Our results showing complete disruption of magnetic compass orientation by broadband electromagnetic noise are in agreement with the results of Ritz et al. (2004) and Engels et al. (2014). The lack of disruption of magnetic compass orientation by the narrow-band electromagnetic fields contradicts the results of similar experiments done with European robins in Frankfurt (Ritz et al., 2004, 2009; Thalau et al., 2005; Wiltschko et al., 2014). More specifically, while we may detect a slight interference but not complete disruption in these fields, we do not see a specifically sensitive effect at ~1.3 MHz, the Larmor frequency of a free electron. However, as described in the introduction, this is also not expected in any biologically and physically plausible radical pair, and certainly not in the currently considered flavin-tryptophan radical pair in cryptochromes (Maeda et al., 2008; Liedvogel and Mouritsen, 2010; Hore, 2012; Solov’yov et al., 2012, 2014).

One main difference between our study and the Frankfurt studies is that all our experiments were performed truly double blind on several levels. Several students evaluated our basic behavioral results blindly and independently, and we used an electromagnetically extremely quiet environment achievable only in our newly constructed laboratory. This means that our birds were only exposed to the one artificially generated time-dependent magnetic field. Hence, we can be sure that there were no background electromagnetic fields from the environment that could have disturbed our birds. In contrast, the Frankfurt experiments were conducted in wooden huts situated within a big city, close to university laboratories. The lack of electromagnetic screening in these huts means that the Frankfurt birds would inevitably have experienced irregular and unpredictable time-dependent fields resulting from the use of electrical equipment in the adjacent buildings. It is unclear what effect this background noise had on the behavioral experiments conducted in Frankfurt.

A recent study done on garden warblers, *Sylvia borin*, at the Courish Spit near Rybachy in Russia (Kavokin et al., 2014) also found a disruptive effect of a 190 nT field with a frequency of 1.403 MHz (the Larmor frequency of a free electron spin in the geomagnetic field of Rybachy). Kavokin et al. (2014) did not screen the background noise, but at the rural location where their experiments were performed, the electromagnetic background noise is likely to have been low. Their results are therefore very interesting. However, to properly interpret their data, it would be important to know whether the effect they observed is specific to the Larmor frequency, or whether they would have seen the same effect if they had used other single frequencies or broadband noise. Furthermore, they only tested each bird three times, so it is difficult to say whether the lack of significant orientation in the group exposed to narrow-band electromagnetic fields truly indicates a complete disruption or if more tests with more birds would have shown a reduction rather than a disruption of their magnetic compass orientation capabilities (reduced directionality vs. full disruption). It will be interesting to see what further experiments with garden warblers at this location will show. Finally, it is important to mention that there may be frequencies other than the Larmor frequency at which a given cryptochrome-based radical pair is sensitive to narrow-band electromagnetic fields and that these frequencies are not necessarily the same in different bird species. It is (at least theoretically) possible that the hyperfine interactions in a given radical pair give rise to resonances which by chance approximately coincide with the Larmor frequency and which could be hit by a narrow-band time-dependent field centered at the Larmor frequency. Likewise, the hyperfine interactions in a given radical pair could give rise to resonances, which by chance could be hit by a different narrow-band time-dependent field in the high kHz to low MHz range. Since cryptochromes in different bird species have different amino acid sequences and are therefore likely to have slightly different hyperfine interactions, such a coincidence would not necessarily be expected for all species.

In conclusion, we could not replicate the previously reported, highly specific, resonance effect on the orientation capabilities of European robins (Ritz et al., 2009). The absence of a sensitive response at the Larmor frequency is not too surprising given that biologically plausible radicals will almost certainly have multiple hyperfine interactions and should therefore not be affected much more strongly at the Larmor frequency than at any other frequency in the low megahertz range. Such radicals would be expected to respond, albeit weakly, to the wide range of frequencies that make up the broadband, noise-modulated electromagnetic fields employed in our study. Nevertheless, it is still not understood how such weak broadband electromagnetic fields could interfere with the radical-pair mechanism (Engels et al., 2014). However, our meticulously collected double-blind experimental data, acquired in a highly electromagnetically screened environment and evaluated independently by several researchers (Engels et al., 2014 and the present study), indicate that weak broadband oscillating fields seem to be much more disruptive to the magnetic compass orientation capabilities of night-migratory European robins (*Erithacus rubecula*) than most if not all strong, narrow frequency band, time-dependent electromagnetic fields.

## Author Contributions

Designed and helped construct the new research laboratory and their screening chambers: HM, N-LS, TR, SS. Designed and supervised the experiments: HM, PJH, SS, N-LS, TR, NB. Performed the experiments: SS, TR, NL, SE, N-LS, DD. Analyzed the data: SS. Wrote the article: SS, HM, PJH. All other authors read and commented on the manuscript. All authors approved the final version to be published.

## Funding

This work was supported by the Defense Advanced Research Projects Agency (QuBE: N66001-10-1-4061 to HM and PJH), the European Research Council (under the European Union’s 7th Framework Programme, FP7/2007-2013/ERC Grant agreement No. 340451 to PJH), the Air Force Office of Scientific Research (Air Force Materiel Command, USAF Award No. FA9550–14–1–0095 to PJH and HM), German Federal Ministry of Education and Research (BMBF; “Varying Tunes”, 01 GQ 0962 to HM), Deutsche Forschungsgemeinschaft (DFG MO1408/1-2 and GRK 1885 to HM) and the Volkswagenstiftung (Lichtenberg Professur to HM).

## Conflict of Interest Statement

The authors declare that the research was conducted in the absence of any commercial or financial relationships that could be construed as a potential conflict of interest.

## Abbreviations

${\text{O}}_{2}^{•-}$: superoxide
NMF: Normal Magnetic Field directed towards the natural magnetic north pole
CMF: Changed Magnetic Field = a magnetic field turned horizontally by −120° from the natural magnetic north pole
SD: standard deviation
mN: magnetic North
gN: geographic North.
